# Supplementary material for: The ubiquitin ligase PHR promotes directional regrowth of spinal zebrafish axons
Source: Commun Biol. 2019 May 22;2:195. doi: 10.1038/s42003-019-0434-2 (PMC6531543; doi:10.1038/s42003-019-0434-2)
Supplement: Supplementary file 1 — Supplementary Information [file 42003_2019_434_MOESM1_ESM.pdf]

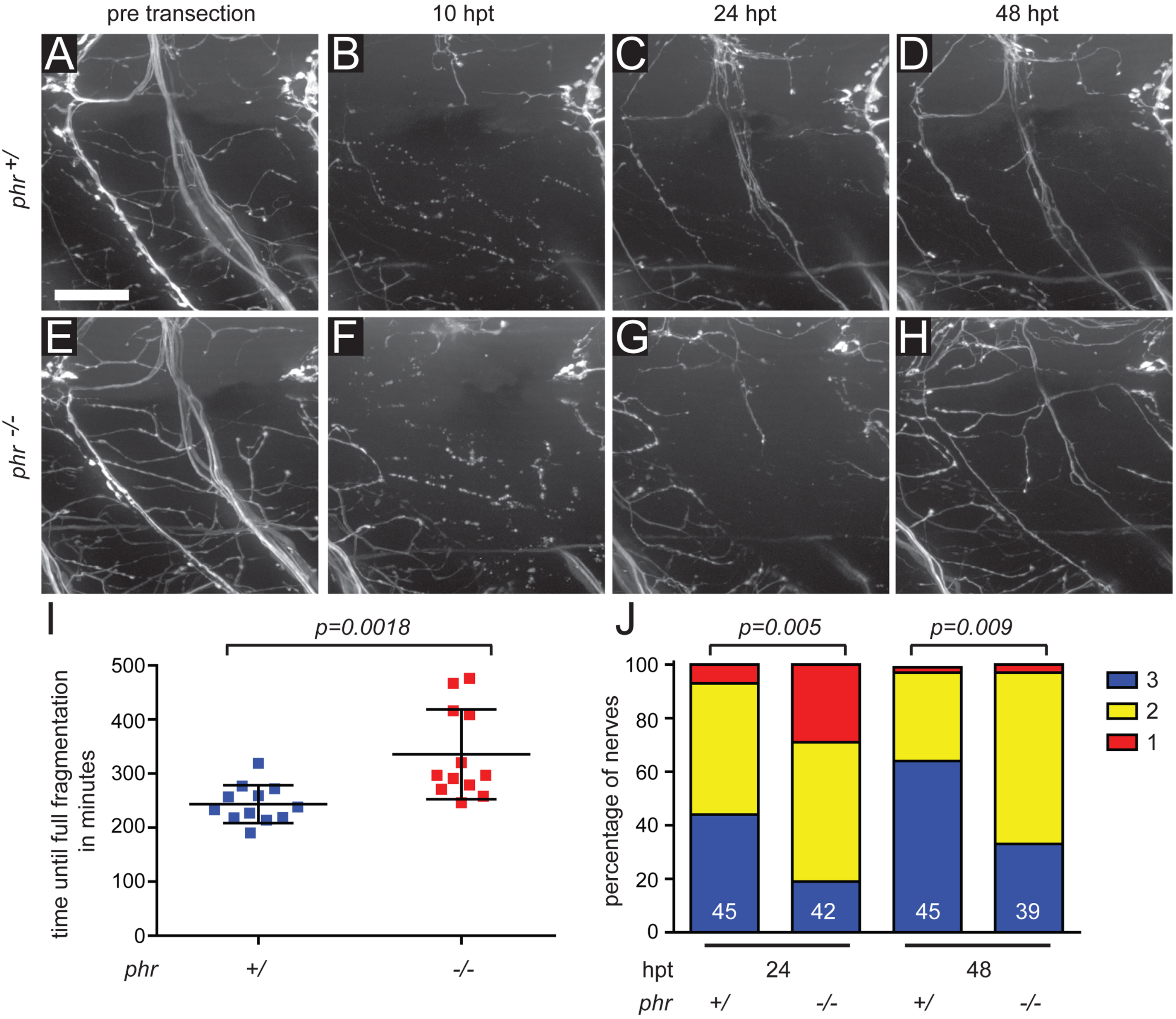


**Supplementary figure 1. PHR controls extent of regrowth and time of Wallerian degeneration in peripheral nerves.** A-J Motor axons in peripheral nerves labeled by the *Tg(mnx1:GFP)* transgene were laser-transected at 5 dpf. Images show a non-mutant sibling (A-D) and a *phr* mutant (E-H); before transection (A,E), full fragmentation of the distal nerve stump at 10 hpt (B,F), some axonal regrowth at 24 hpt (C,G), and increased axonal regrowth at 48 hpt (D,H). Scale bar in A for A-H is 30 µm. We determined the time until full fragmentation by time-lapse imaging (I), showing that Wallerian degeneration was significantly delayed by 37% in *phr* mutants compared to non-mutant siblings. Extent of regrowth was quantified at 24 and 48 hpt (J), the graphical representation of the extent of nerve regeneration (no/ weak, moderate or strong regeneration) is shown, demonstrating that PHR is also required in peripheral nerves for the extent of axon regrowth. No obvious difference in misdirected regrowth was observed. Number of nerves analyzed were: n=12 non-mutant sibling and n=12 *phr* mutant nerves in I; as displayed as white numbers in the bar diagram in J. P-values were determined using a two-tailed student's t-test (I) or the Fischer exact test (J).

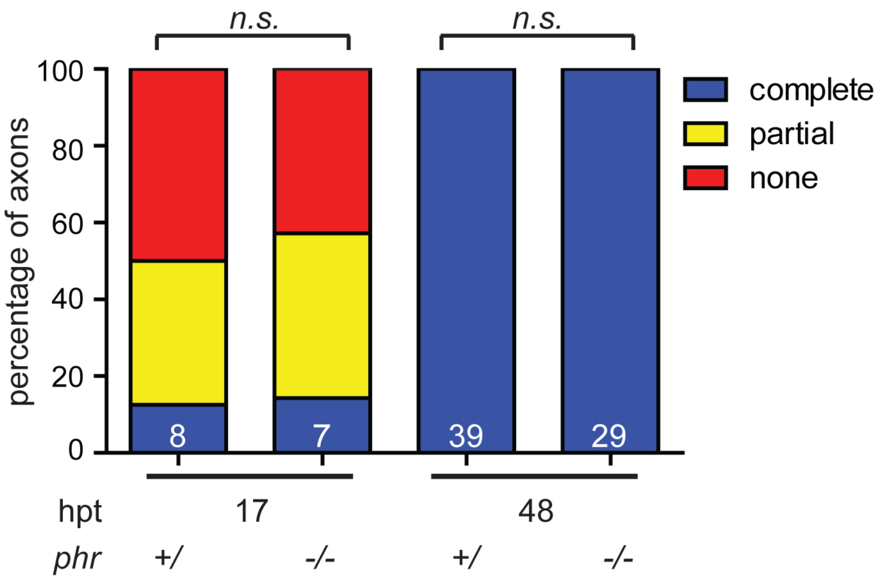


**Supplementary figure 2. PHR does not control Wallerian degeneration of Mauthner axons.** Quantification of Mauthner axon Wallerian degeneration at 17 and 48 hpt is shown. Fragmentation was scored as complete =  along the entire axon, partial = some fragmentation, but not the entire axon was fragmented, none = no axon fragmentation. N=8 non-mutant siblings and n=7 *phr* mutant larvae were analyzed at 17 hpt; n=39 non-mutant siblings and n=29 *phr* mutant larvae were analyzed at 48 hpt. P-values were determined using Fischer exact test. No significant difference was observed.

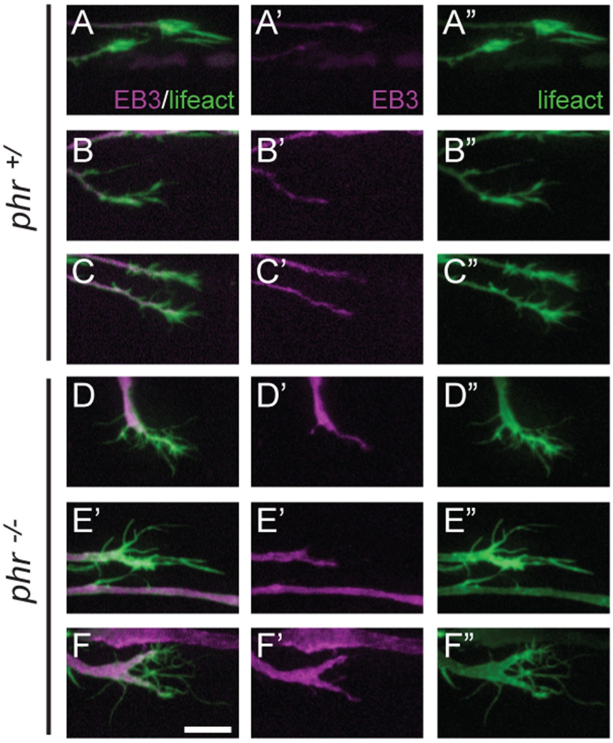


**Supplementary figure 3. Additional examples and individual channels of growth cones in *phr* mutants and non-mutant siblings.** F-actin (green) and microtubules (magenta) simultaneously labeled in growth cone of regrowing Mauthner axons in double transgenic *Tg(hspGFF62a) Tg(UAS:lifeact-GFP-v2a-EB3-RFP)* non-mutant siblings (A-C) or *phr* mutants (D-F). Both channels are shown in A-F, EB3-RFP alone is shown in A'-F' and Lifeact-GFP alone is shown in A"-F". Scale bar in F for all images is 10 µm.

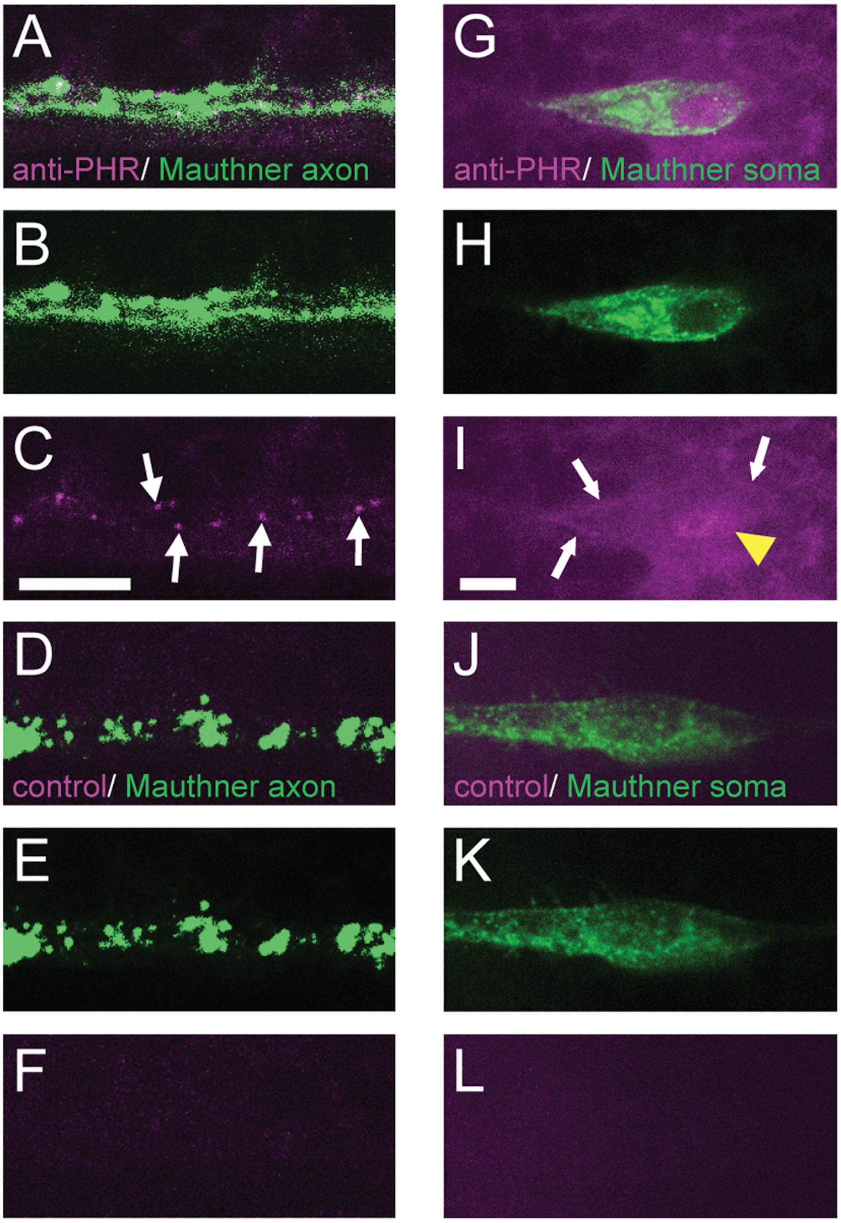


**Supplementary figure 4. PHR is expressed in Mauthner neurons.** Anti-PHR antibody labeling in Tg(hspGFF62a) and Tg(UAS:gap431-20-citrine) double transgenic larvae resulted in a punctate signal along the Mauthner axon (A-C, white arrows in C) and a mainly nuclear but also cytoplasmic signal in the soma of Mauthner neurons (G-I, nuclear signal marked by yellow arrowhead, outline of the soma marked by white arrows). This distribution is in line with the neuronal localization previously reported for PHR [1, 2, 3]. No signal was seen when larvae were incubated with the secondary antibody only for control, neither along the axon (D-F) nor in the Mauthner neuronal soma (J-L). Images in A-F are single planes, images in G-L are maximum projection images of 3 µm stacks. Scale bars in C for A-F and in I for G-L are 10 µm.

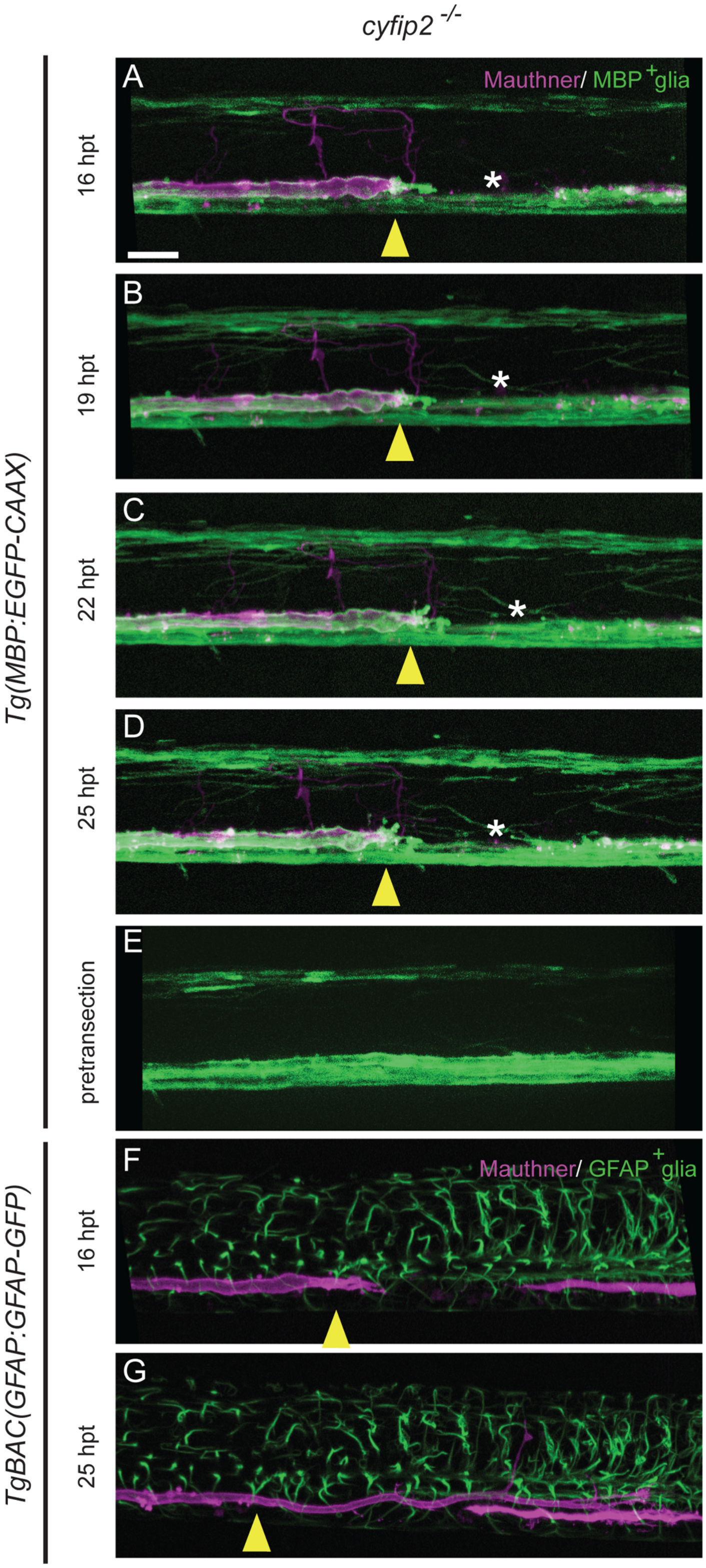


**Supplementary figure 5. *Cyfip2* does not control the morphology of MBP- and GFAP-positive glial cells.** Time-lapse imaging over 9 hours of regrowing Mauthner axons labeled by *Tg(hspGFF62a)* and *Tg(UAS:gap431-20-RFP)*. Myelinating oligodendrocytes are transgenically labeled in *Tg(MBP:EGFP-CAAX)* in A-E and GFAP positive glial cells are labeled in *TgBAC(GFAP:GFAP-GFP)* in F-G. In a *cyfip2* mutant laser-mediated axon transection caused minor damage of myelinating oligodendrocytes (white stars) around the transection site (yellow arrowheads), compared to the pretransection image (E). Except minor damage, myelinating oligodendrocytes neither displayed obvious morphological changes nor interfered with the transection site or formed any obvious scar tissue in n=3 *cyfip2* mutants. The *cyfip2* mutant axon did not regrow over time (A-E). F-G Following laser-mediated axon transection, except minor damage at the transection site (yellow arrowhead), GFAP-positive glial cells neither displayed obvious morphological changes nor interfered with the transection site or formed any obvious scar tissue in n=3 *cyfip2* mutants. This *cyfip2* mutant axon showed some axonal regrowth (F-G). Scale bar in A for A-G is 20 μm.

# Supplementary References


1. Murthy V*, et al.* Pam and its ortholog highwire interact with and may negatively regulate the TSC1.TSC2 complex. *J Biol Chem* **279**, 1351-1358 (2004).

2. Lewcock JW, Genoud N, Lettieri K, Pfaff SL. The ubiquitin ligase Phr1 regulates axon outgrowth through modulation of microtubule dynamics. *Neuron* **56**, 604-620 (2007).

3. Hendricks M, Mathuru AS, Wang H, Silander O, Kee MZ, Jesuthasan S. Disruption of Esrom and Ryk identifies the roof plate boundary as an intermediate target for commissure formation. *Mol Cell Neurosci* **37**, 271-283 (2008).
