# Supplementary material for: The ubiquitin ligase PHR promotes directional regrowth of spinal zebrafish axons
Source: Commun Biol. 2019 May 22;2:195. doi: 10.1038/s42003-019-0434-2 (PMC6531543; doi:10.1038/s42003-019-0434-2)
Supplement: Supplementary file 2 — Description of Supplementary Items [file 42003_2019_434_MOESM2_ESM.pdf]

Supplementary Movie 1. Retraction and membrane sealing at the transection site. Time-lapse movie taken of the transection site during the first 135 min after transection. The axon separates into proximal and distal part, proximal and distal end retract and the membrane reseals. The scale bar is 30 µm.

Supplementary Movie 2. Wallerian degeneration of the distal stump. Time-lapse movie of the distal stump from 20 to 30 hpt, showing axonal fragmentation known as Wallerian degeneration around 29 hpt. The scale bar is 30 µm.

Supplementary Movie 3. Growth cone cytoskeleton in a regrowing non-mutant axon. Time-lapse movie over approximately 10 min, showing the well organized structure of a growth cone in a non-mutant sibling. F-actin labeled by lifeact-GFP and microtubules labeled by EB3-RFP in a regrowing Mauthner axon.

Supplementary Movie 4. Growth cone cytoskeleton in a regrowing *phr*-mutant axon. Time-lapse movie over approximately 10 min, showing a large *phr*-mutant growth cone with several long filopodia. F-actin labeled by lifeact-GFP and microtubules labeled by EB3-RFP in a regrowing Mauthner axon.

Supplementary Data 1. Source data used to generate the plots.
