# Supplementary material for: The ubiquitin ligase PHR promotes directional regrowth of spinal zebrafish axons
Source: Commun Biol. 2019 May 22;2:195. doi: 10.1038/s42003-019-0434-2 (PMC6531543; doi:10.1038/s42003-019-0434-2)
Supplement: Supplementary file 4 — Reporting Summary [file 42003_2019_434_MOESM4_ESM.pdf]

nature research

Corresponding author(s): Juliane Bremer

Last updated by author(s): Apr 5, 2019

Reporting Summary

Nature Research wishes to improve the reproducibility of the work that we publish. This form provides structure for consistency and transparency in reporting. For further information on Nature Research policies, see Authors & Referees and the Editorial Policy Checklist.

Statistics

For all statistical analyses, confirm that the following items are present in the figure legend, table legend, main text, or Methods section.

n/a	Confirmed	
☐	☒	The exact sample size (*n*) for each experimental group/condition, given as a discrete number and unit of measurement
☐	☒	A statement on whether measurements were taken from distinct samples or whether the same sample was measured repeatedly
☐	☒	The statistical test(s) used AND whether they are one- or two-sided<br>*Only common tests should be described solely by name; describe more complex techniques in the Methods section.*
☒	☐	A description of all covariates tested
☐	☒	A description of any assumptions or corrections, such as tests of normality and adjustment for multiple comparisons
☐	☒	A full description of the statistical parameters including central tendency (e.g. means) or other basic estimates (e.g. regression coefficient) AND variation (e.g. standard deviation) or associated estimates of uncertainty (e.g. confidence intervals)
☒	☐	For null hypothesis testing, the test statistic (e.g. *F*, *t*, *r*) with confidence intervals, effect sizes, degrees of freedom and *P* value noted<br>*Give P values as exact values whenever suitable.*
☒	☐	For Bayesian analysis, information on the choice of priors and Markov chain Monte Carlo settings
☒	☐	For hierarchical and complex designs, identification of the appropriate level for tests and full reporting of outcomes
☒	☐	Estimates of effect sizes (e.g. Cohen's *d*, Pearson's *r*), indicating how they were calculated

Our web collection on statistics for biologists contains articles on many of the points above.

Software and code

Policy information about availability of computer code

Data collection	The following software programs were used: Slidebook (3i) and Visiview (Visitron).<br>No custom made codes were used for this study.
Data analysis	The following software programs were used: ImageJ/FIJI (NIH), Graph Pad software (GraphPad). Images were stitched using Photoshop CS (Adobe). No custom made codes were used for this study.

For manuscripts utilizing custom algorithms or software that are central to the research but not yet described in published literature, software must be made available to editors/reviewers. We strongly encourage code deposition in a community repository (e.g. GitHub). See the Nature Research guidelines for submitting code & software for further information.

Data

Policy information about availability of data

All manuscripts must include a data availability statement. This statement should provide the following information, where applicable:

- Accession codes, unique identifiers, or web links for publicly available datasets
- A list of figures that have associated raw data
- A description of any restrictions on data availability

We declare that all the data supporting the findings of this study are available within the paper and the supplementary information files.

Field-specific reporting

Please select the one below that is the best fit for your research. If you are not sure, read the appropriate sections before making your selection.

☒ Life sciences ☐ Behavioural & social sciences ☐ Ecological, evolutionary & environmental sciences

Life sciences study design

All studies must disclose on these points even when the disclosure is negative.

Sample size	For all conditions tested, we first performed a pilot experiment with a minimum of 5 larvae per genotype/ group. In some instances this already resulted in significant regeneration defects. If a non-significant trend towards a regeneration defect was noticed, the sample size was increased until it was clear whether there was a real or a random difference. No statistical method was used to predetermine sample size because we could not predict whether there would be a regeneration defect or the type or severity of the defect.
Data exclusions	For determining the regrowth direction, we only included those axons which formed a growth cone. Where we therefore excluded axons from the analysis of directionality of regrowth, it is stated in the manuscript. All axons however were included in the quantification of extent of regrowth.
Replication	All attempts at replicaton were successful.
Randomization	Larvae used for experiments were selected based on transgene expression and when present on genotype-dependent morphology, i.e. morphological phenotype in mutant larvae. If applicable (e.g. pharmacological treatment), larvae were randomly distributed between experimental and control groups.
Blinding	Most mutant larvae tested had a morphological phenotype and were preselected from clutches based on phenotype. Since larvae had to be mounted under the microscope, they were visually inspected before microscopy, a procedure which cannot be blinded. Therefore blinding was not possible during laser-mediated transections and posttransection microscopy. However, the same transection parameters were applied to all larvae, independent of genotype/ phenotype. Image analyses were performed on images with number codes and no associated genotype information (blinded). Genotype information was revealed after quantification.

Reporting for specific materials, systems and methods

We require information from authors about some types of materials, experimental systems and methods used in many studies. Here, indicate whether each material, system or method listed is relevant to your study. If you are not sure if a list item applies to your research, read the appropriate section before selecting a response.

Materials & experimental systems

n/a	Involved in the study	
☐	☒	Antibodies
☒	☐	Eukaryotic cell lines
☒	☐	Palaeontology
☐	☒	Animals and other organisms
☒	☐	Human research participants
☒	☐	Clinical data

Methods

n/a	Involved in the study	
☒	☐	ChIP-seq
☒	☐	Flow cytometry
☒	☐	MRI-based neuroimaging

Antibodies

Antibodies used	Anti-Phr antibody PP1 (rabbit polyclonal antibody directed against human Phr (aa 4519–4534)) Murthy et al. 2004.
Validation	Using the anti-Phr antibody, we observed a mainly nuclear but also cytoplasmic signal in the soma of Mauthner neurons and a punctate staining along the axon. This distribution is in line with the localization previously reported for Phr (Murthy et al. 2004, Lewcock et al. 2007, Hendricks et al. 2008).<br>No signal was seen in the secondary only control. Using protein BLAST (NIH), we find that parts of the antigenic peptide sequence share ≥ 80% similarity with 3 different regions of zebrafish Phr.

Animals and other organisms

Policy information about studies involving animals; ARRIVE guidelines recommended for reporting animal research

Laboratory animals	This study was performed using larval zebrafish (Danio rerio). The sex in larval zebrafish is not yet determined.<br>The following strains were used: Tg(hspGFF62a), Tg(UAS:gap431-20-citrine) p210Tg, Tg(UAS:gap431-20-RFP), Tg(UAS:lifeact-GFP-v2A-EB3-RFP), cyfip2 p400, celsr3 fh339, dync1h1 hi3684Tg, mycbp2 tn207b, mycbp2 tp203, cfl1l sa5863, dicer1 hu896, dgcr8 fh344, slow learner p174, lrp4 p184, nf1aΔ5 and nf1b+10, pappaa p170, rb1 te226a, robo2 ti272z, sox10 m241, tnc sa1576, TgBAC(GFAP:GFAP-GFP) zf167tg, Tg(MBP:EGFP-CAAX) ue2tg, Tg(mnx1:GFP) ml2
Wild animals	The study did not include wild animals.
Field-collected samples	The study did not involve samples collected from the field.

Ethics oversight	Protocols and procedures involving zebrafish were in compliance with the University of Pennsylvania Institutional Animal Care and Use Committee (IACUC) regulations.

Note that full information on the approval of the study protocol must also be provided in the manuscript.
